# Daratumumab binds to mobilized CD34+ cells of myeloma patients in vitro without cytotoxicity or impaired progenitor cell growth

**DOI:** 10.1186/s40164-018-0119-4

**Published:** 2018-10-16

**Authors:** Xun Ma, Sandy W. Wong, Ping Zhou, Chakra P. Chaulagain, Parul Doshi, Andreas K. Klein, Kellie Sprague, Adin Kugelmass, Denis Toskic, Melissa Warner, Kenneth B. Miller, Lisa Lee, Cindy Varga, Raymond L. Comenzo

**Affiliations:** 1 The John C Davis Myeloma and Amyloid Program in the Division of Hematology-Oncology, Department of Medicine, Tufts Medical Center, 800 Washington Street, Box 826, Boston, MA 02111 USA; 2 Division of Hematology and Blood and Marrow Transplantation, Department of Medicine, University of California, San Francisco, CA USA; 3 Taussig Cancer Institute of Cleveland Clinic, Maroone Cancer Center, Weston, FL USA; 4 Janssen Research & Development, Spring House, PA USA

**Keywords:** Myeloma, Daratumumab, CD34+, Progenitor cells

## Abstract

**Background:**

The monoclonal antibody daratumumab, approved for treating myeloma, targets CD38, a protein on myeloma and also on CD34+ hematopoietic progenitor cells. Because mobilized CD34+ cells are critical for stem cell transplant, we investigated the in vitro activity of daratumumab on mobilized CD34+ cells from myeloma patients with no prior exposure to daratumumab.

**Methods:**

We determined the number of CD38 molecules per CD34+ cell, and whether daratumumab bound to CD34+ cells, whether C1q bound to daratumumab-coated CD34+ cells and whether daratumumab-related complement-dependent cytotoxicity (CDC) occurred. We also examined CD34+ cell progenitor cell colony capacity in assays with pre-plating incubation of CD34+ cells with daratumumab alone or with daratumumab and the CD59 inhibitory antibody BRIC229, and also assessed CD34+ cell responses to increasing doses of daratumumab in caspase 3/7 activity assays.

**Results:**

Although 75% of mobilized CD34+ cells co-express CD38, CD38 was minimally present on CD34+ cells compared to Daudi and KG-1 controls, C1q did not bind to daratumumab-coated CD34+ cells, and CDC did not occur. CD34+ cells incubated in complement-rich human serum with daratumumab alone or with daratumumab and BRIC229, and then plated in progenitor cell assays, produced similar numbers of colonies as controls. In progenitor cell assays with cryopreserved or fresh unselected or CD34-selected cells, daratumumab did not affect progenitor cell capacity, and in caspase 3/7 activity assays CD34+ cells were not affected by increasing doses of daratumumab.

**Conclusion:**

In vitro, daratumumab is not toxic to mobilized CD34+ progenitor cells from myeloma patients.

## Background

CD38 is a type II membrane protein active in receptor-mediated adhesion, calcium mobilization, formation of cyclic ADP-ribose (ADPR) from nicotinamide adenine dinucleotide (NAD^+^), and hydrolysis of cADPR into ADP-ribose [1–3]. CD38 also mediates activation and proliferation of lymphocytes and regulates extracellular NAD^+^ levels [4]. Over several decades, monoclonal antibodies to CD38 had been developed for use against hematological malignancies without success until the identification of daratumumab, a monoclonal anti-CD38 approved for myeloma in late 2015 [5–8].

Daratumumab’s mechanisms of action include complement-dependent cytotoxicity (CDC), antibody-dependent cellular cytotoxicity (ADCC), antibody-dependent phagocytic cytotoxicity (ADPC) and enzymatic interference triggering apoptosis. CD38 is also found on normal human marrow and mobilized hematopoietic progenitor cells, particularly lineage committed CD34+ cells, where its expression is responsive to various cytokines [9–11]. In view of the role of autologous SCT in patients with multiple myeloma [12], we investigated CD38 expression on mobilized CD34+ cells from myeloma patients and the binding and effect of daratumumab on mobilized CD34+ cells in vitro.

## Methods

### Patients and cells

On an IRB approved study requiring informed consent, myeloma patients undergoing SCT (none of whom had ever been treated with daratumumab) donated mobilized blood cells for research, used fresh after collection or thawed from cryopreserved products. Patients were mobilized with G-CSF and plerixafor and cells collected by leukapheresis. Cells were used after Ficoll-Pague separation or CD34+ cell selection with MiniMACS (Miltenyi Biotec, Auburn, CA). Controls were Daudi, IM-9 and KG-1 cells from American Type Culture Collection (Manassas, VA) cultured as directed.

### Antibodies and flow cytometry

Daratumumab was from Janssen Pharmaceuticals (Titusville, NJ), isotype control (human IgG1 kappa) from Sigma-Aldrich (St Louis MO), and anti-CD38-APC, anti-CD34-PerCP, anti-CD59-FITC (H19 clone) and isotype controls from BioLegend (San Diego, CA). Second antibody for daratumumab binding was mouse anti-human IgG Fc APC-conjugated (HP6017, BioLegend). The anti-C1q was a rabbit polyclonal FITC-conjugated (Abcam, Cambridge, MA) used with an appropriate isotype control. BRIC 229, a CD59 neutralizing antibody, was obtained from the International Blood Group Reference Laboratory of the Bristol Institute for Transfusion Sciences (NHS Blood and Transplant, Bristol, UK), and the anti-CD46 monoclonal GB24 was kindly provided by Dr. J. Aktinson, Washington University, St. Louis, MO, USA. Antibodies were titrated for optimal use and analyses performed on a BD Accuri flow cytometer (BD Biosciences, San Jose, CA).

### CD38 quantitation and daratumumab binding assay

The phycoerythrin (PE) fluorescence quantitation kit Quantibrite™ with anti-CD38-PE (clone HB7), both from BD, were used to estimate the number of cell-surface CD38 molecules by flow cytometry. For daratumumab binding studies, we incubated the cells with 2.5 µg/mL daratumumab or human IgG_1_ kappa isotype control, and then stained with mouse anti-human IgG Fc or control and analyzed them by flow cytometry.

### Complement-dependent cytotoxicity (CDC)

Complement-rich human serum (CRHS) was from Innovative Research (Novi, MI), was aliquoted, cryopreserved and thawed for immediate use. For CDC studies, cells were aliquoted at 4 × 10^5^ per well, incubated in 10% complement-rich serum with daratumumab or isotype control at 1 µg/mL for 15 min at room temperature, then for 1 h at 37° C in 5% CO_2_, and then were washed, resuspended with 5 μg/mL propridium iodide (PI, Sigma-Aldrich) and analyzed by flow cytometry [13]. In these and other studies the doses of daratumumab used in vitro were based on the activity defined for daratumumab in assays against human myeloma cells [14]. For C1q binding studies, we used the same steps of incubation and washing, then stained with either the FITC-conjugated rabbit polyclonal anti-human C1q or isotype control. For BRIC 229 and GB24 studies we incubated with BRIC 229 or GB24, washed and resuspended, and then CDC in response to daratumumab was analyzed as above.

### Progenitor cell assays

Progenitor cell assays (PCA) (Stem Cell Technologies, Vancouver, CA; Cat #04435) were performed according to manufacturer’s instructions. Fresh or thawed unselected cells at 5 × 10^4^/mL and CD34-selected cells at 500/mL medium were plated in triplicate. In some experimental situations we incubated prior to plating with daratumumab, BRIC229, isotype control or combinations for 1 h at 37° C in 5% CO_2_ and then added CRHS and incubated for another hour before plating directly into methylcellulose medium without washing the cells. Results of at least three experiments with specimens from different patients are reported. PCA were coded and counted on day 14 by a blinded investigator.

### Caspase 3/7 activity

Luciferin-based caspase 3/7 activity (Promega; Madison, WI) was evaluated following manufacturer’s instructions on a Promega GloMax microplate luminometer with 5 × 10^3^ cells per well, reported as relative luminescence units (RLU).

### Statistics

Descriptive statistics, analyses and plots were performed with MedCalc Statistical Software version 17.9.2 (MedCalc Software, Ostend, Belgium; http://www.medcalc.org; 2017) and with GraphPad Prism version 5.00 for Windows (GraphPad Software, San Diego California USA, http://www.graphpad.com). All assays were performed at least three times unless otherwise noted.

## Results

### Patients and cells

Specimens of mobilized peripheral blood CD34+ cells were obtained from 16 patients, 11 of whom were men, with a median age of 58 (range 50–69). All patients received bortezomib and dexamethasone with either cyclophosphamide or lenalidomide, and underwent stem cell mobilization a median of 8 months (3–18) after diagnosis with myeloma. None had received daratumumab or elotuzumab prior to mobilization and all were mobilized with G-CSF and plerixafor.

**Fig. 1:**
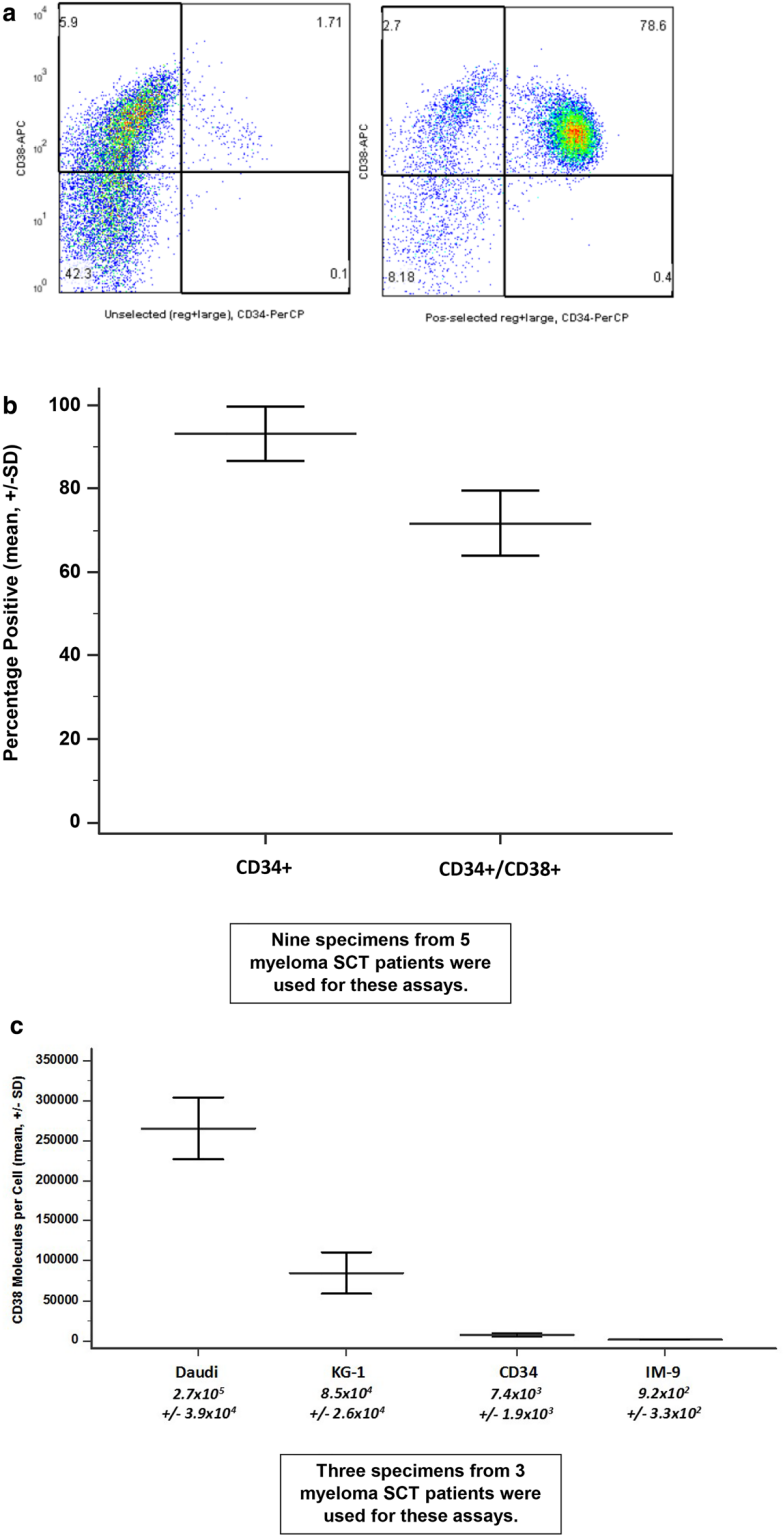
Fewer CD38 molecules are present on mobilized CD34+ cells compared to Daudi and KG-1 cells. **a** This is a representative flow cytometry scatter plot of CD34 and CD38 positivity on cells from a mobilized unselected cell suspension (left) and a CD34-selected suspension (right), showing that CD34+ cells express CD38+ as has been previously described [18]. **b** This graph shows the mean (±SD) numbers of CD34+ and CD34+/CD38+ cells in 9 specimens of mobilized blood CD34-selected cells from 5 myeloma patients, indicating that the purity of the selected specimens and the fraction of double positive cells were both high. **c** With the BD Quantibrite™ assay, we determined the mean number of CD38 molecules per CD34+ mobilized cell from myeloma patients and used as controls Daudi, KG-1 and IM-9 cells. Daudi and KG-1 cells have over 35- and tenfold more CD38 molecules per cell compared to CD34+ mobilized cells from myeloma patients

**Fig. 2:**
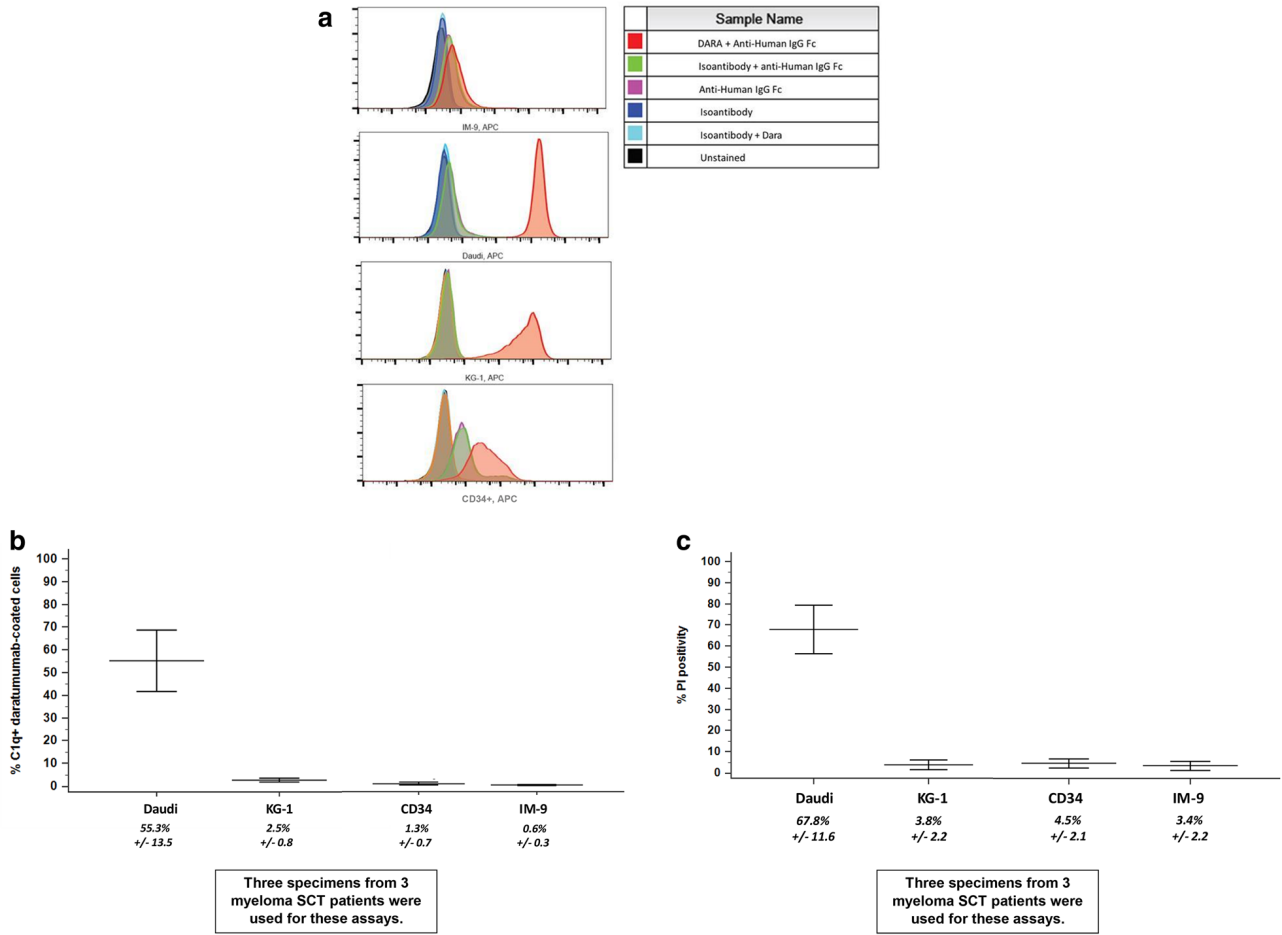
Daratumumab binds to CD34+ cells but C1q does not. Daratumumab does not induce complement-dependent cytotoxicity of CD34+ cells in vitro. **a** CD34+ cells and the control cells were incubated with daratumumab and its binding was confirmed for < 1% of IM-9, 99% of Daudi, 96% of KG-1, and 40% of CD34+ cells; the isotype control bound to 5% of the CD34+ cells. This is a representative flow plot with controls as designated. **b** After incubation with complement-rich human serum, C1q binding was assessed. C1q was found on Daudi but was negligible on the other cells (mean ± SD). **c** When the density of CD38 is sufficient, the binding of daratumumab is also adequate for formation of the membrane attack complex by C1q, permitting CDC to occur [16]. As this graph depicts, daratumumab-mediated CDC occurs with Daudi and minimally with KG-1 but not with CD34+ or IM-9 cells (mean ± SD, n = 3)

The yields of CD34-selected cells from each specimen varied, and the cell numbers required by the different experimental studies varied also. Therefore, each specimen was used for one or a limited number of specific studies. From the 16 patients we obtained 21 specimens. Nine specimens from 5 patients were used to determine the number of CD38 molecules on CD34-selected cells (Fig. 1), and three specimens from 3 patients for assessment of daratumumab, C1q binding and CDC (Fig. 2). Three specimens from 2 patients were used fresh for CD34-selected (Fig. 4) and for thawed unselected progenitor cell assays (Fig. 4a). Three specimens from 3 patients were used for assays resulted in Fig. 5b and another 3 specimens from 3 patients were used for assays resulted in Fig. 5c. Those 6 specimens were also used in the caspase 3/7 assays resulted in Fig. 5d.

**Fig. 3:**
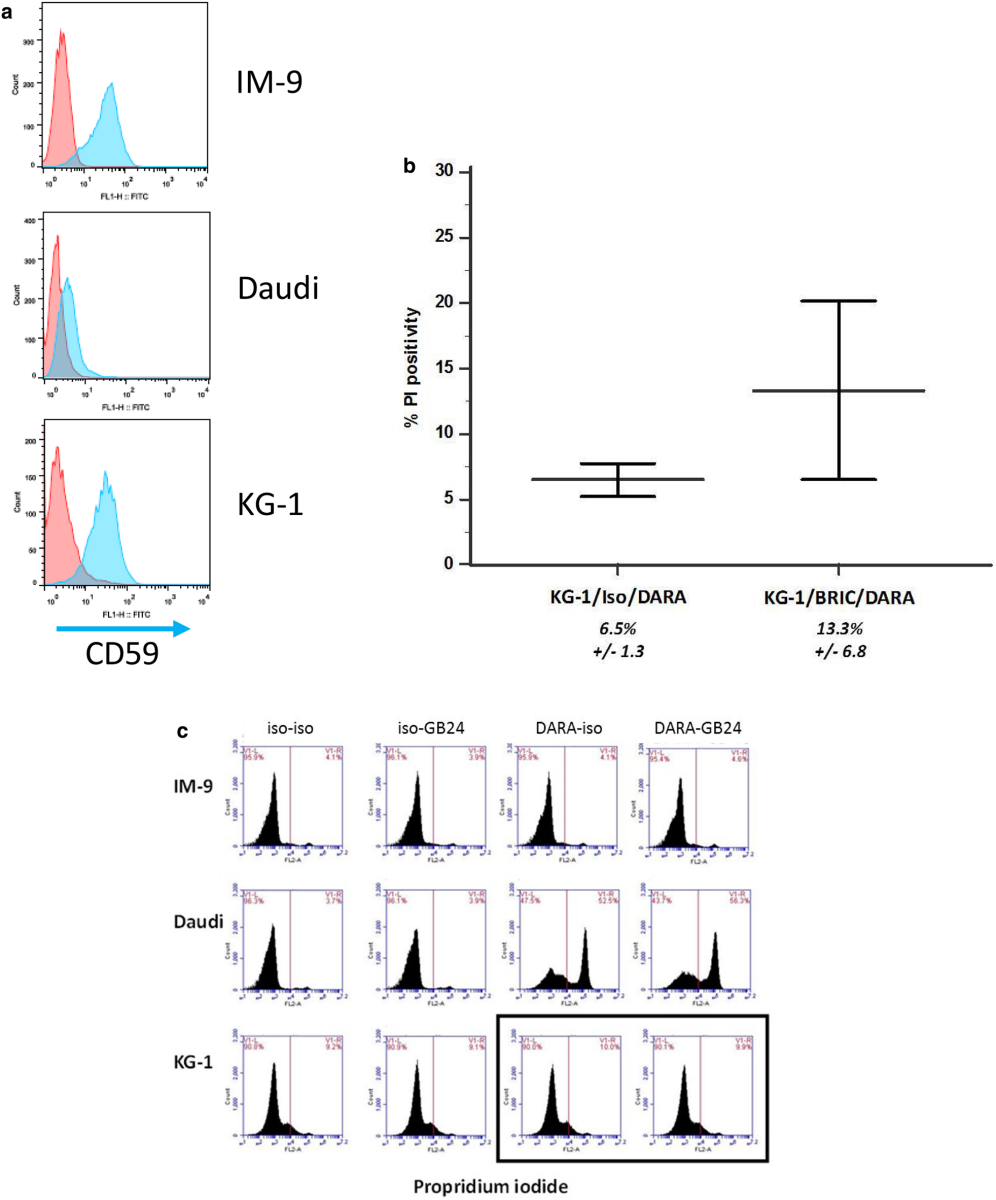
CD59 is present on IM-9 and KG-1 cells but not on Daudi cells. With CD46 inhibition daratumumab does not cause CDC of KG-1 cells and with CD59 inhibition it does not impair the colony-forming capacity of CD34+ cells. **a** CD59 expression (blue) is high on IM-9 and KG-1 cells but not on Daudi cells. **b** When the BRIC229 antibody that inhibits CD59 is used with KG-1 cells in daratumumab CDC, the level of PI positivity increases minimally (mean ± SD). **c** When the GB24 antibody that neutralizes CD46 is used, there is no increase in PI positivity as highlighted (box) for KG-1, a stand-in for CD34+ cells, or other cells in this representative plot

**Fig. 4:**
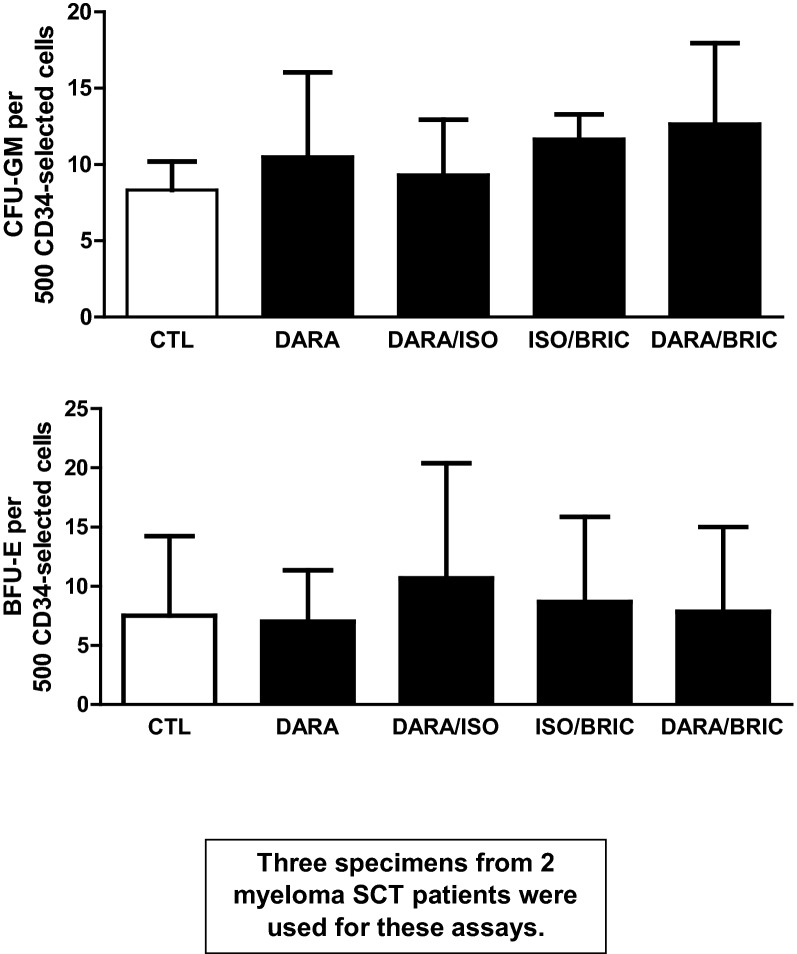
CD34-selected cells were incubated for several hours with daratumumab, BRIC229 or both and then plated in progenitor cell assays to assess their function. Pre-incubation did not diminish colony production compared to controls (mean ± SD)

**Fig. 5:**
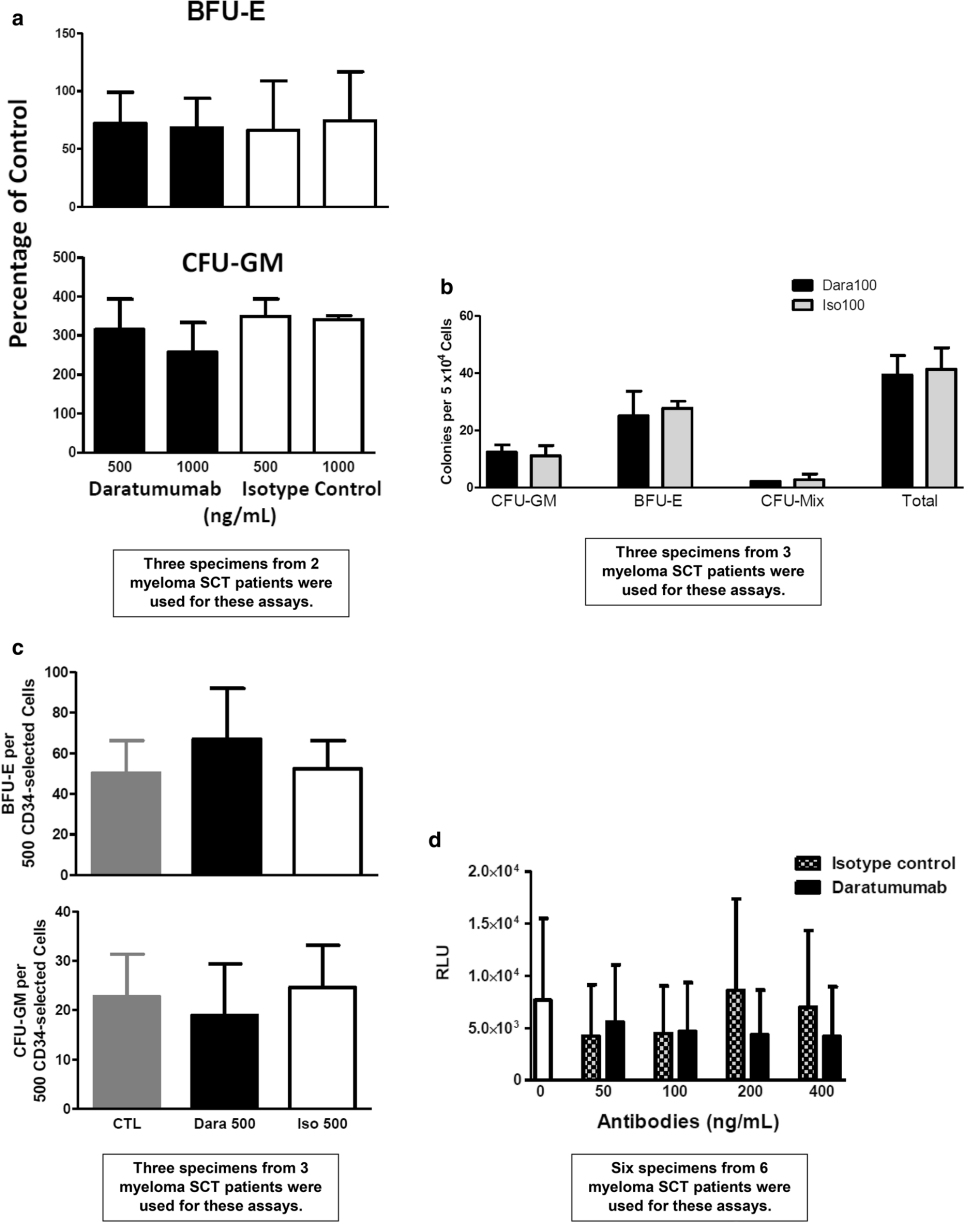
Daratumumab does not impair colony production by thawed or fresh mobilized CD34+ progenitor cells or cause apoptosis of CD34+ cells in vitro. **a** Cryopreserved thawed unselected mobilized human blood progenitor cells were plated at a concentration of 5 × 10^4^ cells/mL of semisolid medium with daratumumab or isotype control at 500 and 1000 ng/mL, and colonies were counted on day 14 (mean ± SD). There was no decrease in CFU-GM or BFU-E with daratumumab. **b** Fresh unselected mobilized peripheral blood progenitor cells were plated in semisolid medium containing daratumumab or isotype control (100 ng/mL) at 5 × 10^4^ cells/mL and no difference was seen in colony counts (mean ± SD). **c** CD34-selected cells were incubated in complement-rich human serum with no antibody, daratumumab or isotype control at 500 ng/mL for 1 h and then plated directly in semisolid medium at 500 cells/mL. There were no significant decreases in colonies with daratumumab compared to isotype control (mean ± SD). **d** CD34-selected cells were incubated in complement-rich human serum with no antibody, daratumumab or isotype control in increasing amounts and then assessed for evidence of caspase 3/7 activity (RLU, relative light units) (n = 6) (mean ± SD). There was no evidence of caspase 3/7 activity at any concentration of daratumumab used

### CD38 molecules on CD34+ cells

We determined that approximately 75% of CD34+ mobilized peripheral blood progenitor cells from myeloma patients en route to SCT co-expressed CD38 (Fig. 1a, b), and then sought to determine the number of CD38 molecules per CD34+ cell. Although CD38 was expressed on many CD34+ mobilized cells (Fig. 1b), the number of C38 molecules per CD34+ (or IM-9) cell was significantly lower than the number per Daudi or KG-1 cell respectively (Fig. 1c). Daudi cells have over 250,000 molecules of CD38 per cell on their surface while KG-1 cells have 85,000 per cell and CD34+ mobilized cells from myeloma patients have less than 10,000 per cell.

### Daratumumab binding and C1q docking

We then asked whether daratumumab bound to Daudi, IM-9, KG-1 and CD34+ cells, whether CDC occurred and if C1q docked. After incubation with daratumumab and control, daratumumab could be found on Daudi, KG-1 and CD34+ cells but not on IM-9 cells (Fig. 2a). CDC occurred with two-thirds of daratumumab-coated Daudi cells but with less than 5% of each of the others (Fig. 2b), likely because antigen density was not adequate for sufficient antibody binding and C1q docking. Additional studies determined that, while 55% of daratumumab-coated Daudi cells permitted C1q docking, very few of the other cells did (Fig. 2c), consistent with a role for antigen density in CDC. Without C1q bound to the cell surface appropriately, the complement cascade cannot proceed and CDC cannot occur.

### CD59 and CD46 expression and activity

CD59 and CD46 are membrane complement regulatory proteins; the former inhibits the formation of the membrane attack complex by preventing C9 binding while the latter is a co-factor that inactivates C3b and C4b [15]. We examined CD59 and CD46 expression, confirming that CD59 and CD46 expression was high on KG-1 and IM-9 but very low on Daudi cells (Fig. 3a shows CD59 expression). We found CDC with three antibodies and beads coating CD34-selected cells technically problematic in the CD59 blocking assays with BRIC229; the difference in this regard between CD34+ cells and the cell lines we used is reflected in Fig. 2a where isotype control bound to CD34+ cells at a low level, possibly reflecting steric events associated with residual changes of CD34-selection. Therefore, for this assay, we used KG-1 cells as a stand-in for CD34+ cells. KG-1 cells have higher levels of CD38 on the cell surface than CD34-selected cells (Fig. 1c) and yet the increase in CDC activity was minimal with BRIC229 to neutralize CD59; daratumumab-related mean CDC was minimally increased with KG-1 cells from 6.5 to 13.3% (Fig. 3b). When GB24 was used blocking CD46, there was no effect on CDC with Daudi, IM-9 and KG-1 cells (Fig. 3c).

To evaluate the functional impact of CD59 inhibition on CD34-selected cells, we performed colony-forming assays with CD34-selected cells from mobilized leukapheresis products, pre-incubating them first with BRIC229 and/or daratumumab and then with CRHS for a total of 2 h, and plating them directly into the semisolid medium. We saw no difference in colony numbers with daratumumab or BRIC229 alone or BRIC229 with daratumumab compared to controls (Fig. 4).

### Progenitor cell and apoptosis assays

We also performed a series of progenitor cell assays with both thawed and fresh mobilized peripheral blood cells (Fig. 5a, b), unselected and CD34-selected (Fig. 5b, c), to examine further the impact of daratumumab on colony-forming capacity. There were no differences with respect to colony-forming capacity with any of these cell suspensions between daratumumab and controls. We note that fresh unselected mobilized blood cells contain potential effector cells so the lack of a difference in progenitor cell growth with daratumumab in culture suggests that ADCC did not occur to any significant degree. Furthermore, in bioluminescent caspase 3/7 activity assays with increasing doses of daratumumab and CRHS, we did not observe apoptosis of CD34-selected cells (Fig. 5d).

## Discussion

Antibody-mediated complement-dependent cytotoxicity (CDC) depends on target antigen levels, antibody affinity and isotype, complement membrane regulatory proteins (cMRP; CD46, CD55, CD59), and the localization of sufficient bound antibody to permit docking of C1q [13, 16]. Therefore, after confirming that approximately 75% of CD34+ mobilized peripheral blood progenitor cells from myeloma patients en route to SCT co-expressed CD38 (Fig. 1a, b), we sought to determine the number of CD38 molecules per CD34+ cell. Although CD38 was expressed on many CD34+ mobilized cells (Fig. 1b), the number of C38 molecules per CD34+ (or IM-9) cell was significantly lower than the number per Daudi or KG-1 cell respectively (Fig. 1c).

We then asked whether daratumumab bound to these cells, whether C1q docked on daratumumab coated cells, and whether CDC using complement-rich human serum (CRHS) occurred. After incubation with daratumumab and control, daratumumab could be found on Daudi, KG-1 and CD34+ cells but not on IM-9 cells (Fig. 2a). Moreover, 55% of daratumumab-coated Daudi cells permitted C1q docking, while very few IM-9 or KG-1 cells did (Fig. 2b). CDC occurred with two-thirds of daratumumab-coated Daudi cells but with less than 5% of each of the others (Fig. 2c), likely because antigen density was not adequate for sufficient antibody binding and C1q docking. Although the differences we describe are related primarily to CD38 expression on the surface of Daudi, IM-9 and KG-1 cells, the cells we used were not synchronous prior to testing, and therefore cell-cycle related variables creating subpopulations may have contributed to the activity levels we observed.

Daratumumab then does not significantly impact the viability or colony-forming capacity of CD34+ progenitor cells from myeloma patients in vitro. We show this for cryopreserved and fresh unselected as well CD34+ cells (Fig. 5), and also show that in vitro caspase 3/7 activity is not increased in CD34+ cell suspensions with increasing doses of daratumumab in complement-rich human serum (Fig. 5d). Nevertheless, our claims are limited in this report because not all mechanisms of daratumumab activity were extensively examined and the number of observations is small. Our claims also require further study in part because additional possibilities exist. Just as daratumumab treatment can modulate CD38 and cMRP expression on myeloma cells, it may be that long-term exposure to daratumumab leads to attenuation of CD38 expression on CD34+ cells, perhaps affecting mobilization kinetics and lineage-specific progenitor cell frequencies or proliferative capacity, particularly when daratumumab is combined with other agents [13]. Hypothetically, such effects may be linked not only to altered CD38 expression on CD34+ cells but also to changes in migration signaling and in the hematopoietic niche. Nevertheless, it is important to note that we and others recently reported that six myeloma patients induced with lenalidomide, carfilzomib and daratumumab, who proceeded to post-induction mobilization and SCT, had a median of 20 × 10^6^ CD34+ cells per kg (range 6.5–38) collected with cytokine mobilization after a median of 4.5 cycles of induction (4–9), and that these 6 patients then underwent SCT with clinical outcomes and hematopoietic recoveries typical for myeloma SCT [17].
